# Phase I study of novel SYK inhibitor TAK‐659 (mivavotinib) in combination with R‐CHOP for front‐line treatment of high‐risk diffuse large B‐cell lymphoma

**DOI:** 10.1002/jha2.625

**Published:** 2022-12-07

**Authors:** Reem Karmali, Frederique St‐Pierre, Shuo Ma, Kelly D. Foster, Jason Kaplan, Xinlei Mi, Barbara Pro, Jane N. Winter, Leo I. Gordon

**Affiliations:** ^1^ Robert H. Lurie Comprehensive Cancer Center, Division of Hematology/Oncology, Feinberg School of Medicine Northwestern University Chicago Illinois USA; ^2^ Division of Hematology/Oncology Northwestern University Chicago Illinois USA; ^3^ Northwestern Medicine Lake Forest Hospital Lake Forest Illinois USA; ^4^ Department of Preventative Medicine ‐ Biostatistics, Feinberg School of Medicine Northwestern University Chicago Illinois USA

**Keywords:** aggressive non‐Hodgkin's lymphoma, diffuse large B‐cell lymphoma, mivavotinib, SYK inhibitor, TAK‐659

## Abstract

**Background**: TAK‐659, a novel oral SYK inhibitor, has demonstrated efficacy in heavily pretreated diffuse large B‐cell lymphoma (DLBCL). We report results of a phase I single‐institution escalation study of front‐line treatment with R‐CHOP and TAK‐659 in treatment‐naïve high‐risk DLBCL.

**Methods**: Patients with high‐risk DLBCL were treated with R‐CHOP for 1 cycle, followed by combined R‐CHOP and TAK‐659 for an additional five cycles, with TAK‐659 dosing escalated from 60 mg, to 80 mg, to 100 mg daily, based on a 3 + 3 design. The primary objective was to determine the safety and establish the maximum tolerated dose (MTD) of TAK‐659 in this setting.

**Results**: Twelve patients were enrolled. Dose level 3 (100 mg) was established as the MTD. Dose level 1 (60 mg) maintained a similar area under the curve (AUC) to the MTD. With a median follow‐up of 21 months, 92% of patients achieved complete response (CR). The most common treatment‐emergent adverse events were lymphopenia (100%), infection (50%, *n* = 3 opportunistic), aspartate aminotransferase elevation (100%), and alanine aminotransferase elevation (83%).

**Conclusion**: A TAK‐659 dose of 60 mg was well tolerated, did not require dose modifications, and maintained a similar AUC to the MTD. The combination of R‐CHOP and TAK‐659 in patients with newly diagnosed high‐risk DLBCL produces promising CR rates.

## 1 INTRODUCTION

Diffuse large B‐cell lymphoma (DLBCL) is the most common type of non‐Hodgkin lymphoma (NHL), with approximately 20,000 new cases diagnosed annually in the US. [1]. DLBCL is an aggressive subtype of NHL and is invariably fatal without treatment [2]. The standard treatment for patients with newly diagnosed DLBCL is chemoimmunotherapy with R‐CHOP (rituximab, cyclophosphamide, doxorubicin, vincristine, prednisone) [3]. Treatment with R‐CHOP results is an estimated 2‐year and 5‐year progression free survival (PFS) of 75% and 65%, respectively, and an estimated 2‐year and 5‐year overall survival (OS) of 80% and 75%, respectively [3]. DLBCL is highly heterogeneous in its underlying biology and clinical behavior [4, 5]. Several high‐risk disease features and poor prognostic factors are associated with a higher propensity for refractory disease or relapse after standard R‐CHOP therapy [6]. Known high‐risk features include the activated B cell (ABC)/non‐germinal center B cell (non‐GCB) cell of origin subtype [7, 8], rearrangement or overexpression of the human MYC oncogene [9, 10], and transformation of a low‐grade/indolent lymphoma to a DLBCL histology after treatment of the indolent lymphoma with an anthracycline‐based therapy [11, 12]. Each of these features is associated with a 5‐year OS of approximately 50% or less [7, 8, 9, 10, 11, 12]. In addition to these disease‐specific factors contributing to a worse prognosis, prognostic models that also account for patient‐specific factors, such as the NCCN‐IPI, also help predict higher risk patients. Using the National Comprehensive Cancer Network‐International Prognostic Index (NCCN‐IPI), patients with DLBCL treated in the rituximab era with a high‐intermediate risk score of 4–5 and a high‐risk score of ≥6 had a 5‐year OS of 64% and 33%, respectively [13].

There is a significant need for more effective therapies in the front‐line setting in patients with high‐risk DLBCL. TAK‐659, or mivavotinib, is an orally (PO) bioavailable, potent and reversible inhibitor of spleen tyrosine kinase (SYK) and FMS‐like tyrosine kinase 3 (FLT3), and is currently under development for the treatment of patients with advanced malignancies [14, 15, 16, 17]. SYK is a nonreceptor protein tyrosine kinase with SH2‐binding domains that bind to phosphorylated immunoreceptor tyrosine activation motifs (ITAMs) located in B cells, T cells, and natural killer (NK) cells. SYK becomes activated upon ITAM binding and subsequently controls the activity of downstream signaling pathways such as phosphoinositide 3‐kinase (PI3K), mitogen‐activated protein kinase, and nuclear factor kappa‐B (NF‐kB), which mediate cell proliferation, differentiation, and survival. The SYK pathway is implicated in the pathogenesis of hematologic and solid tumors [18, 19].

TAK‐659 has demonstrated inhibitory activity in preclinical models of DLBCL, follicular lymphoma (FL) [20], chronic lymphocytic leukemia [21], and Epstein‐Barr virus (EBV)‐associated lymphoma [22, 23]. A phase I trial evaluated the safety of TAK‐659 in 105 patients with relapsed/refractory (R/R) B‐cell lymphoma or solid tumors. Among the 43 patients with R/R DLBCL, the overall response rate (ORR) was 28%, with a complete response (CR) rate of 19%. Responses were seen in both de novo and transformed disease and occurred in both non‐GCB and GCB subgroups. The maximum tolerated dose (MTD) was 100 mg, and treatment was well tolerated with the most common treatment‐emergent adverse events (TEAEs) being asymptomatic and reversible elevations in clinical laboratory values [24].

On this basis of this preclinical and clinical data, we conducted a phase I study of TAK‐659 in combination with R‐CHOP to determine the safety, tolerability, and MTD/recommended phase II dose (RP2D) of TAK‐659 when utilized with chemoimmunotherapy for the front‐line treatment of high‐risk DLBCL.

## 2 MATERIALS AND METHODS

### 2.1 Study design

This was a phase I, single‐center, open‐label, single‐arm dose‐escalation study of TAK‐659 in combination with R‐CHOP. The primary objective was to determine the safety and establish the MTD of TAK‐659 when combined with R‐CHOP in the front‐line treatment of high‐risk DLBCL. Secondary objectives included assessments of preliminary efficacy of this combination as determined by ORR by positron emission tomography‐computed tomography (PET‐CT) (Lugano 2014 criteria), PFS, OS, as well as the pharmacokinetic (PK) profiles of TAK‐659 according to dose. Our exploratory objective was to characterize the PK of TAK‐659 in combination with R‐CHOP.

Inclusion criteria were age ≥18 years, ECOG 0–2, and untreated stage I‐IV DLBCL with high‐risk features defined as one of the following features: ABC/non‐GCB subtype, high‐intermediate or high‐risk NCCN‐IPI (score ≥4), MYC gene rearranged by FISH including double‐hit lymphoma (DHL), double expressing DLBCL (DEL; overexpression of MYC ≥40% AND BCL2 ≥50% by immunohistochemistry respectively), or previously treated transformed low‐grade lymphoma without prior exposure to anthracycline. Patients were excluded if they had known central nervous system (CNS) disease or were HIV positive.

Patients were treated with R‐CHOP for one cycle on or off study followed by combined treatment with R‐CHOP and TAK‐659 for an additional five cycles on study. TAK‐659 was started with cycle 2 to allow for inclusion of patients who required emergent treatment. Additionally, patients could have received a bridge of rituximab or steroids prior to C1 of R‐CHOP. TAK‐659 was dosed daily with dosing escalated from 60 mg (dose level 1), to 80 mg (dose level 2), to 100 mg (dose level 3) based on a 3 + 3 design. The study schema is outlined in Supplemental 1, and treatment administration summary is available in Supplemental 2.

The study was conducted in compliance with the Declaration of Helsinki, International Conference on Harmonization Good Clinical Practice standards, and applicable regulatory requirements. Relevant institutional review boards or ethics committees approved all aspects of the study. All patients provided written informed consent. The trial is registered at ClinicalTrials.gov (NCT03742258).

### 2.2 Statistical design

Patients were enrolled to dose level 1 initially (60 mg) and escalated to 80 mg for dose level 2, and 100 mg for level 3 utilizing a standard “3+3” dose escalation design to identify dose limiting toxicity (DLT) and determine the MTD. Definition of DLT and criteria for determination of MTD are included in Supplemental 3 and Supplemental 4, respectively. A dose level −1 (40 mg) was included in the event that level 1 was determined to be too toxic.

AEs were assessed throughout the study and graded according to the NCI Common Terminology Criteria for AEs version 5.0. Toxicity and tolerability were assessed by history and physical, including vital signs and ECOG performance status, and laboratory values to determine incidence and grade of AEs, serious AEs (SAEs), and DLT. Patients were evaluated for DLT during the first cycle (21 days) of TAK‐659 combined with R‐CHOP (study Cycle 2) to determine the MTD. All patients who receive at least one dose of TAK‐659 were considered evaluable.

ORR, using Lugano criteria (2014), was defined as the percentage of subjects with a confirmed CR or partial response as assessed by the investigators. Response was assessed by comparison of PET/CT after C3 and after completion of treatment to PET/CT at screening. All patients who received at least one dose of TAK‐659, had sites of measurable disease at baseline, and one postbaseline disease assessment were evaluable for this endpoint. PFS was defined as the time from study enrollment until progression/recurrence of lymphoma or death from any cause and was assessed at 12 and 18 months. OS was defined as the time from study enrollment until death from any cause and was also assessed at 12 and 18 months.

## 3 RESULTS

### 3.1 Patient demographics

Twelve patients were enrolled from December 2019 to November 2021. Enrollment was initially affected by the COVID‐19 pandemic, resulting in a 2‐year enrollment period for this trial. The median age was 64 years (range 25–75). Eight (67%) patients had stage III/IV disease, and four (33%) had a high‐risk NCCN‐IPI ≥4. By histology, seven (58%) patients had DEL, three (25%) had transformed FL (tFL), one (8%) had Richter's transformation, and one (8%) had DHL. By cell of origin, of the seven (58%) patients with DLBCL, not otherwise specified (NOS), four were of GCB subtype, and three of ABC/non‐GCB subtype DLBCL. The patient with Richter's transformation had small lymphocytic lymphoma with initial presentation of diffuse lymphadenopathy and no significant leukemic phase, and was treatment‐naïve at the time of transformation. The four patients with early‐stage disease had stage II disease with at least one high‐risk feature. Two patients were age >60, one patient had a high lactate dehydrogenase (LDH) suggestive of extensive disease, and one patient had primary extranodal DLBCL presenting with life‐threatening bowel perforation. Demographic and clinical factors are summarized in Table 1.

**TABLE 1:** Baseline demographics

Demographic parameter	All patients (*n* = 12)
Age (y), median (range)	64 (25–75)
Sex, male, *n* (%)	5 (42)
ECOG, *n* (%)	
0	8 (67)
1	4 (33)
High risk NCCN‐IPI (≥4), *n* (%)	4 (33)
Elevated LDH, *n* (%)	3 (25)
Advanced stage, *n* (%)	8 (67)
Presence of extranodal disease, *n* (%)	5 (42)
Histology at enrollment, *n* (%)	
DLBCL, NOS	7 (58)
Transformed from low‐grade lymphoma*	4 (33)
Double hit lymphoma	1 (8)
MYC aberrancy	
Sole overexpression of MYC	1 (8)
Double expressor (MYC and BCL‐2)	7 (58)
Double hit + double expressor (MYC and BCL‐2)	1 (8)
Cell of origin	
Germinal center	7 (58)
Non‐germinal center	5 (42)
Cycle 1 R‐CHOP off study, *n* (%)	3 (25)

* 1 Richter's and 3 transformed follicular lymphoma.

### 3.2 Efficacy outcomes

**FIGURE 1:**
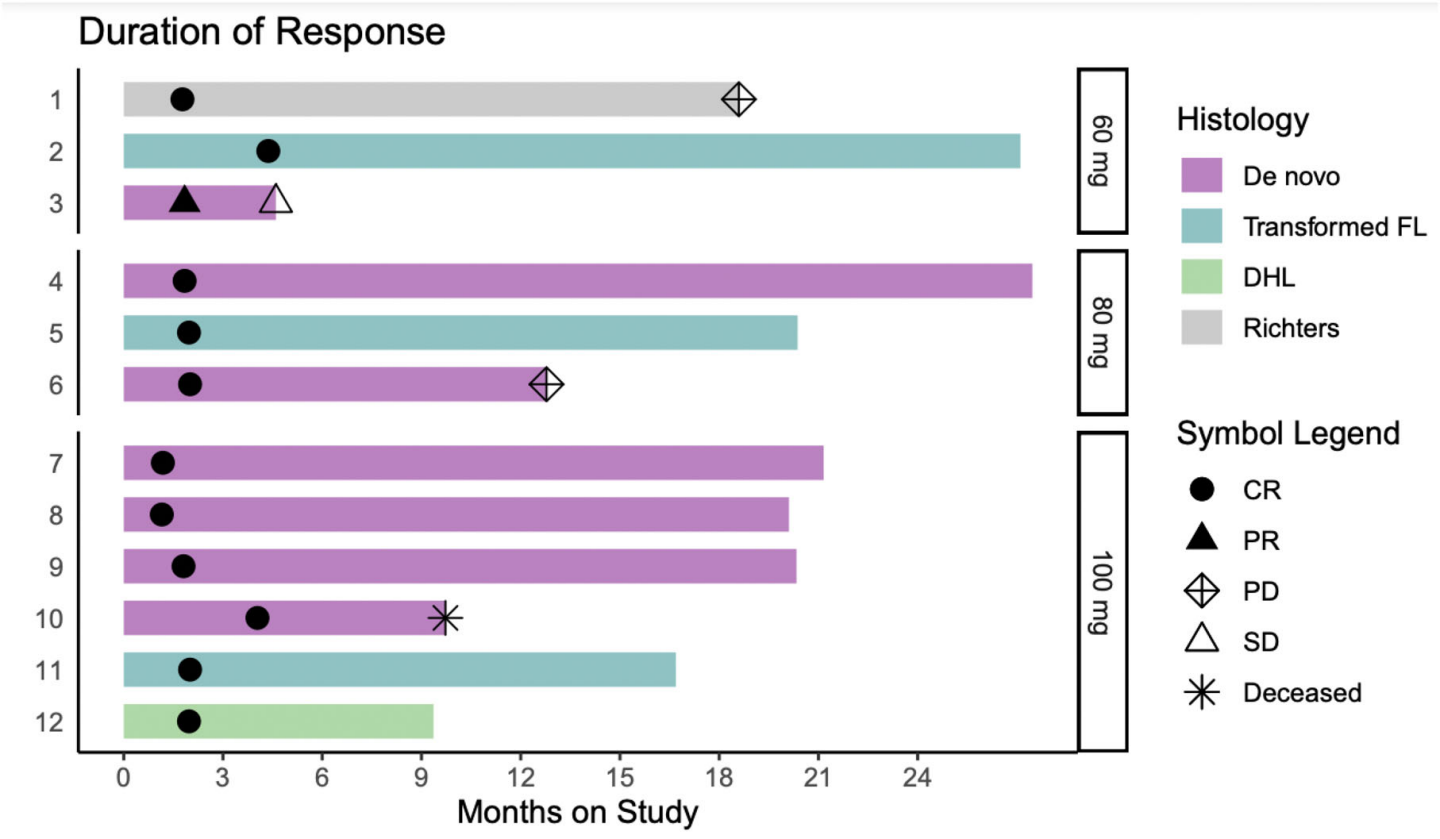
Response assessment/durability of response by dose cohorts

**FIGURE 2:**
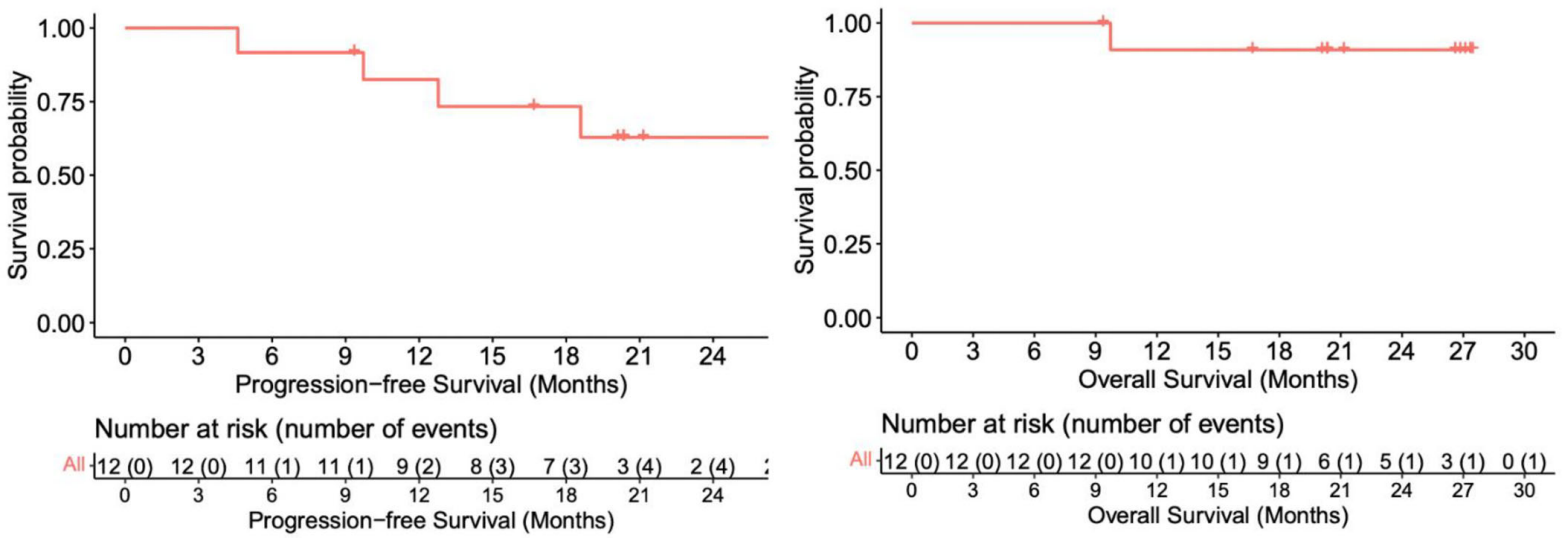
Progression free survival and overall survival in treated cohort

ORR was 100%, with 92% of patients achieving CR. With a median follow‐up of 21 months, one patient had primary refractory disease (ABC and DEL), two patients with CR subsequently progressed (one non‐GC DLBCL, one Richter's), and one patient died of cardiogenic shock unrelated to the study drug. Figure 1 depicts response rates and duration of response according to histologic diagnosis. The CR rate for patients with tFL was 100% (3/3), and 86% (6/7) for patients with DEL. The 12‐, 18‐, and 24‐month PFS rates in all patients were 83%, 73%, and 63%, respectively, and the OS rate was 90% at 24 months (Figure 2).

### 3.3 PK studies and toxicities

**FIGURE 3:**
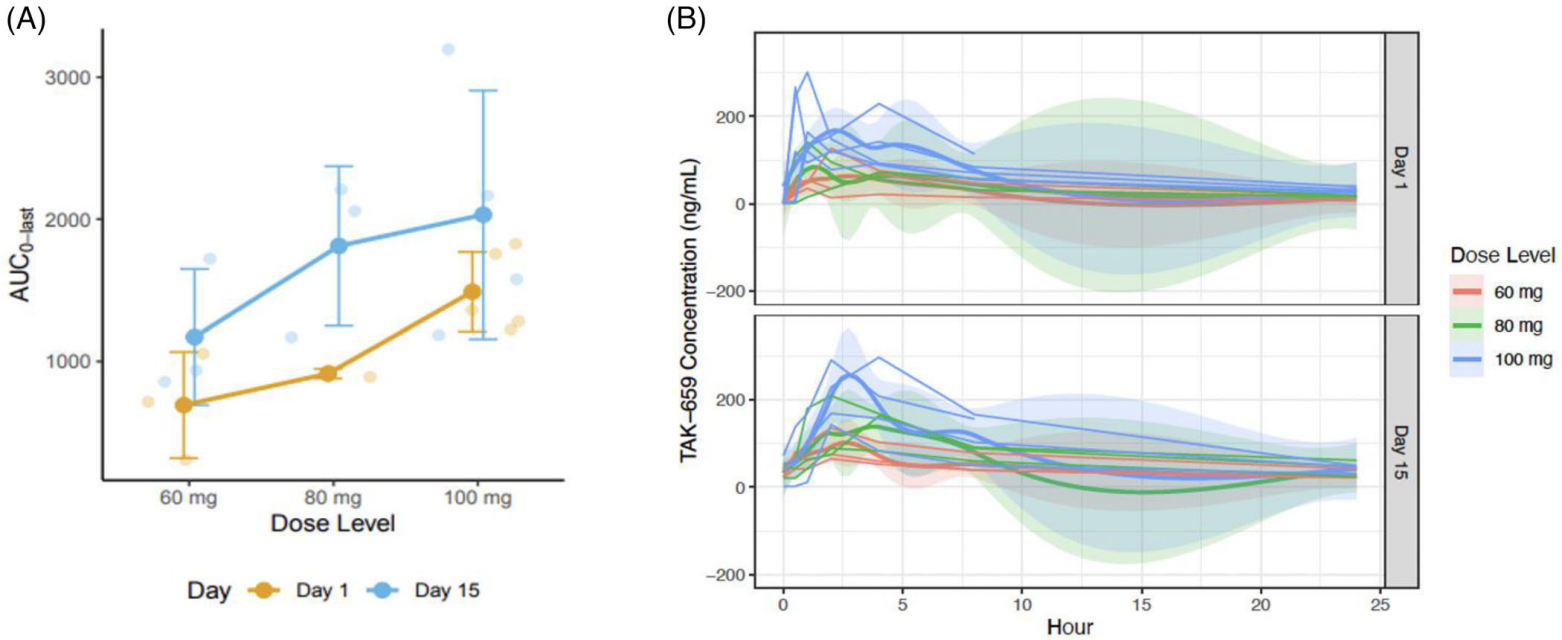
Pharmacokinetics of TAK‐659 when combined with R‐CHOP. (A) Area under the curve (AUC) by dose level. (B) TAK‐659 concentration by time on days 1 and 15

Dose level 3 (100 mg) was established as the MTD. PKs were measured pre‐ and postdose D1 and D15 of cycle 2. Cuzick's test signaled an increase in area under the curve (AUC) by dose level on day 1 (*p* = 0.01) but not by day 15 for 100 mg as compared to 60 or 80 mg (Figure 3). Three patients were treated with the 60 mg dose, three patients with the 80 mg dose, and six patients with the 100 mg dose. Three patients received CNS prophylaxis (two with intrathecal methotrexate, one with high‐dose methotrexate), at the discretion of the investigator.

There was one DLT on dose level 3 (grade 3 hypertension). No DLTs were recorded at dose levels 1 and 2. All TEAEs by dose level are outlined in Table 2. The most common TEAEs attributed to TAK‐659 were lymphopenia (*n* = 12, 100%), infection (*n* = 6, 50%), aspartate aminotransferase (AST) elevation (*n* = 12, 100%), and alanine aminotransferase (ALT) elevation (*n* = 10, 83%). Incidence and severity of transaminase elevation was consistent with prior reports for this agent. The most common grade 3/4 toxicities were hematologic. Median number of cycles of TAK‐659 delivered was 5 (range 3–5). TEAEs led to TAK‐659 dose modifications in 8 (67%) pts, although none at dose level 1: 2 (17%) required permanent dose reductions (both for lung infections), while five (42%) required discontinuation (four for infection and one for febrile neutropenia). R‐CHOP administration was delayed in 2 pts secondary to TAK‐659‐related TEAEs. Aside from dose modifications of vincristine for peripheral neuropathy, no additional dose modifications for R‐CHOP were needed. Infections included bacterial (*n* = 3) and opportunistic infections (*n* = 1 for each pneumocystis jirovecii pneumonia (PJP), cytomegalovirus (CMV), and aspergillosis), and one case of COVID‐19. Growth factor and antimicrobial prophylaxis were not mandated, although all patients received antiviral prophylaxis with acyclovir and growth factor support with pegfilgrastim or tbo‐filgrastim. PJP prophylaxis was advised for CD4 counts <200 at initial diagnosis.

**TABLE 2:** Treatment‐related adverse events of interest from the combination of TAK‐659 and R‐CHOP at each dose level

Adverse event	All patients (*n* = 12)								
Dose level	1 (*n* = 3)			2 (*n* = 3)			3 (*n* = 6)		
Grade	1–2	3	4	1–2	3	4	1–2	3	4
Hematologic									
Lymphopenia	2	1	0	2	0	1	0	2	3
Lymphocytosis	0	0	0	1	0	0	0	0	0
Anemia	1	0	0	3	0	0	2	4	0
Thrombocytopenia	0	0	0	1	0	0	1	2	2
Neutropenia	0	0	0	0	0	0	0	1	3
Febrile neutropenia	0	0	0	0	0	0	0	2	0
Nonhematologic									
AST elevation	2	0	0	3	0	0	4	1	0
ALT elevation	1	0	0	3	0	0	2	0	0
Alk phos elevation	0	0	0	3	0	0	2	0	0
LDH elevation	1	0	0	2	0	0	4	0	0
Bilirubin elevation	1	0	0	0	0	0	0	0	0
Amylase elevation	0	0	0	0	0	0	2	0	0
Lipase elevation	0	0	0	1	0	0	3	0	0
GGT elevation	0	0	0	1	0	0	1	0	0
Hypocalcemia	0	0	0	2	0	0	2	1	0
QTc prolongation	0	0	0	1	0	0	1	1	1
Atrial fibrillation	0	0	0	0	0	0	0	1	0
Infection*	0	0	0	0	2	0	1	2	1
Fever	1	0	0	2	0	0	0	0	0
Peri‐orbital/facial swelling	0	0	0	0	0	0	1	0	0
Fatigue	0	0	0	1	0	0	3	0	0
Weight loss	0	0	0	1	0	0	0	0	0
Anorexia	0	0	0	1	0	0	0	0	0
Myalgia	1	0	0	0	0	0	0	0	0
Arthralgia	0	0	0	1	0	0	0	0	0
HTN	0	0	0	1	1	0	1	0	0
Diarrhea	0	0	0	1	0	0	2	1	0
Nausea	0	0	0	0	0	0	1	0	0
Vomiting	0	0	0	0	0	0	1	0	0

Abbreviations: Alk Phos, alkaline phosphatase; ALT, alanine aminotransferase; AST, aspartate transaminase; HTN, hypertension; LDH, lactate dehydrogenase; PJP, pneumocystis jirovecii; QTc, corrected QT interval.

* Infections included sepsis, pneumonia, conjunctivitis, sinusitis, oral thrush, CMV reactivation, aspergillosis, and PJP pneumonia.

## 4 DISCUSSION

Data from the phase I trial of single‐agent TAK‐659 demonstrated clinically meaningful responses in a heavily treated population of patients with R/R DLBCL, including 19% achieving CR, with overall good tolerability. This response was seen in patients with both GCB and ABC/non‐GCB cell of origin subtypes of DLBCL. Among the responders, 75% (9/12) had a response lasting ≥ 16 weeks, and 33% (4/12) had ongoing response at the data cutoff [24]. Promising results from this trial, including the favorable toxicity profile of TAK‐659 and the lack of significant interaction or overlapping side effects with R‐CHOP, justify the use of TAK‐659 in the front‐line setting in DLBCL, in combination with the standard of care regimen of R‐CHOP.

We describe results of the combination of TAK‐659 with R‐CHOP as first‐line treatment in high‐risk DLBCL. Patients with high‐risk DLBCL have a higher risk of relapsing or being refractory to front‐line treatment with R‐CHOP. Specific high‐risk populations include patients with ABC/non‐GCB subtype [7, 8], DHL or DEL [25, 26, 27], and indolent lymphomas that transform to DLBCL [11, 12]. In patients with transformed lymphoma who previously received treatment for their indolent lymphoma, 2‐year OS is only 39% without consolidation with autologous stem cell transplantation. In those who never received treatment prior to transformation, outcomes are similar to DLBCL, NOS (2‐year OS approximately 80%)[12]. In patients with DEL treated with R‐CHOP, 2‐year OS is estimated at 50% [25], and 5‐year OS is approximately 40% [28].

Albeit a small sample size, our cohort of patients was enriched for DEL (*n* = 7, 58%) and tFL (*n* = 3, 25%), and we found a 12‐month PFS and OS of 83% and 90%, respectively. In comparison to historical OS in these disease states, these results appear promising for this high‐risk patient population. Our cohort also included three patients (25%) with DLBCL, NOS with a non‐GCB cell of origin subtype. The 12‐month PFS of 83% also appears encouraging in comparison with the historical 2‐year PFS of 60% in patients with the non‐GCB subtype of DLBCL.

All patients experienced some degree of elevated transaminases (typically concurrent AST and ALT). The majority (10/12) of patients had a transient LDH elevation. These laboratory abnormalities were asymptomatic and grade <3 with the exception of 1 grade 3 elevation in AST. This is in keeping with previous reported AEs in the phase I trial by Gordon et al.[24] All patients had complete reversal of these abnormal laboratory values after completion of treatment. Dosing was continued uninterrupted in the event of asymptomatic laboratory abnormalities, and no short term or long‐term clinical consequences were noted from these abnormalities. We believe these toxicities should be considered asymptomatic artifacts of treatment and therefore should not inform dose modifications with future evaluation of TAK‐659.

Infection risk may represent a limitation to this regimen. We noted an increased risk of opportunistic infections, with three patients (25%) developing such an infection (PJP, CMV, and aspergillosis, [*n* = 1, each]). Stricter prophylaxis practices were adopted later in the course of the study to mediate this increased risk, resulting in no subsequent cases of opportunistic infections.

It should be noted that the FLYER trial, which evaluated four versus six cycles of R‐CHOP in patients with stage I‐II DLBCL, was published after enrollment for this trial had begun, therefore early‐stage patients were not considered for an abbreviated course of R‐CHOP. Additionally, the early‐stage patients included in our trial were high‐risk and did not fit the inclusion criteria for the FLYER trial (age <60, ECOG status 0–1, normal LDH, nonbulky disease) [29]. The UNFOLDER study, which randomized patients with bulky or extranodal disease to 6 cycles of R‐CHOP with or without radiation and showed no difference in PFS and OS, remains a standard of care in patients with high‐risk features despite limited stage, and provides rationale for our clinical trial design [30].

With respect to dosing of TAK‐659, we note that while the 100‐mg dose was well‐tolerated as a single agent, the dose of 60 mg was better tolerated in combination with chemoimmunotherapy, without requirement for any dose modification, and with no infectious complications encountered at this dose. The dose of 60 mg daily maintained a similar AUC to the MTD of 100 mg after 2 weeks of exposure.

## 5 CONCLUSION

TAK‐659, a novel SYK inhibitor, combined with R‐CHOP in patients with newly diagnosed high‐risk DLBCL including DLBCL transformed from follicular lymphoma and DEL, is well tolerated at a dose of 60 mg, with no requirement for dose modification. A dose of 60 mg maintained a similar AUC to the MTD of 100 mg with ongoing treatment. Opportunistic infections were noted with this treatment combination, suggesting that patients should receive aggressive anti‐microbial prophylaxis to mitigate infection risk. This combination regimen resulted in promising CR rates and survival outcomes at 12 months in this high‐risk population. A larger phase II trial to confirm the efficacy of TAK‐659, in combination with R‐CHOP, would be warranted.

## AUTHOR CONTRIBUTIONS

All authors contributed significantly to the production of the study and manuscript, and all authors have approved the submission of this article.

## CONFLICT OF INTEREST

Dr. Reem Karmali ‐ Advisory Board: Celgene Corporation, Gilead Sciences, Juno Therapeutics, Kite Pharma, Janssen, Karyopharm, Pharmacyclics, Morphosys, Epizyme, Genentech/Roche, EUSA, Calithera; Grants/Research Support to Northwestern: Celgene Corporation/Juno Therapeutics/BMS, Takeda, BeiGene, Gilead Sciences/Kite; Speakers Bureau: AstraZeneca, BeiGene, Gilead Sciences, Morphosys. Dr. Jane Winter ‐ Research Funding to Northwestern: Merck (JNW); Research Funding to University of Chicago: Cellectis (Spouse), Gilead (Spouse), Novartis (Spouse); Honoraria: Merck (JNW); Consultancy: Novartis (Spouse), CVS/Caremark (Spouse); Data Safety Monitoring Board: Novartis (Spouse), Epizyme (Spouse).

## ETHICS STATEMENT

This study was approved by the Northwestern University IRB (STU00207880‐CR0002). Written consent was obtained from all included patients

## Supporting information

Supporting Information

## Data Availability

The data that support the findings of this study are available from the corresponding author upon request.
