# Supplementary material for: Phase I study of novel SYK inhibitor TAK‐659 (mivavotinib) in combination with R‐CHOP for front‐line treatment of high‐risk diffuse large B‐cell lymphoma
Source: EJHaem. 2022 Dec 7;4(1):108–14. doi: 10.1002/jha2.625 (PMC9928783; doi:10.1002/jha2.625)
Supplement: Supplementary file 1 — Supporting Information [file JHA2-4-108-s001.docx]

**Supplemental Materials**

Supplemental 1: Study Schema


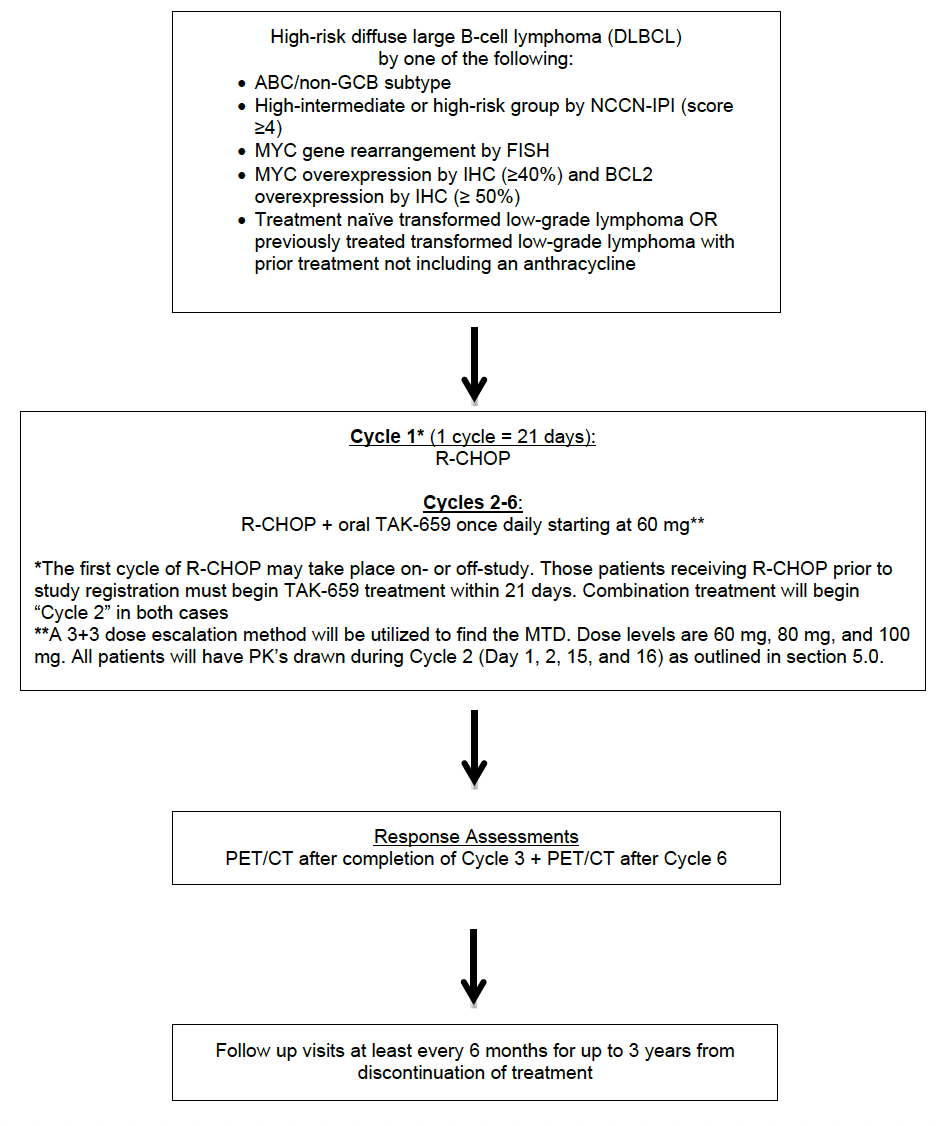


Supplemental 2: Treatment Administration Summary


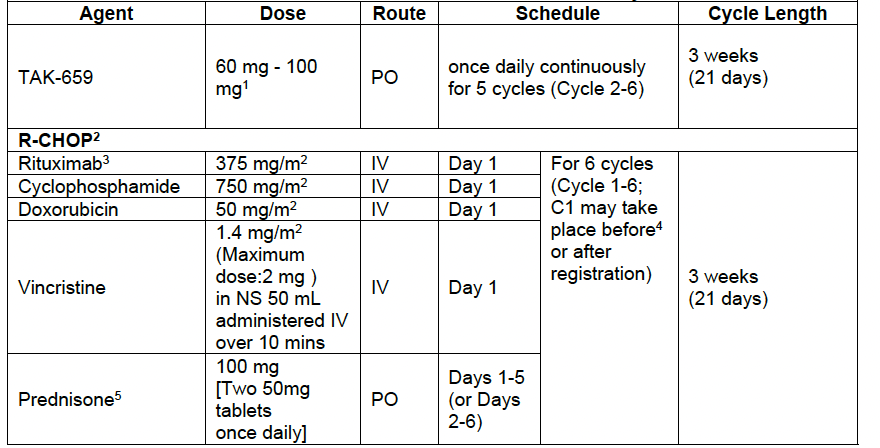


^1^Patients will undergo a 3+3 dose escalation of TAK-659 as detailed in section 4.3. The starting dose will be 60 mg with subsequent dose levels at 80 mg and 100 mg. In the event that the starting dose is determined to be too toxic, a dose level -1 has been included at 40 mg. On days with combination treatment, TAK-659 should be given before starting R-CHOP administration with consideration for pre-medications and TAK-659 PK’s.

^2^Investigator may choose to administer G-CSF (filgrastim/tbo-filgrastim or pegfilgrastim) with Cycle 1 per standard ASCO guidelines. It is also highly recommended for Cycle 2-6 (combination therapy).

^3^Acetaminophen 650 mg PO and diphenhydramine 50-100 mg IV or PO 30-60 minutes prior to rituximab. Permitted to give corticosteroids prior to rituximab (up to 10 mg prednisone equivalent), in addition to the scheduled prednisone as part of R-CHOP, as per the discretion of the investigator.

^4^If R-CHOP is administered off-study prior to registration, TAK-659 treatment must begin ≤30 days after initiating R-CHOP.

^5^On any clinic day (for study treatment), patients should NOT take prednisone before coming to clinic, but should hold and bring the prednisone tablets to clinic.

Supplemental 3: Definitions of Dose Limiting Toxicity

Toxicity will be evaluated according to the NCI CTCAE version 5.0. DLT is defined as any of the events listed below. All adverse events meeting this criteria will be considered DLT’s unless the event is clearly unrelated to study therapy.

1. Grade 4 neutropenia (ANC <500 cells/mm3) unresolved to ≤Grade 1 (ANC >1500 cells/mm3) or baseline for more than 7 consecutive days.
2. ≥Grade 3 neutropenia (ANC <1000 cells/mm3) with fever and/or infection, where fever is defined as an oral temperature ≥38.3°C.
3. Grade 4 thrombocytopenia (<25,000/mm3) unresolved to ≤Grade 1 (>75,000/mm3) or baseline for more than 7 consecutive days or a platelet count <10,000/mm3 at any time.
4. ≥Grade 3 thrombocytopenia (<50,000/mm3) with clinically significant bleeding.
5. Grade 4 anemia.
6. Any Grade 3 or greater non-hematologic toxicity with the following exceptions:
   1. Grade 3 arthralgia/myalgia.
   2. Grade 3 rash lasting ≤7 days with optimal treatment that includes topical steroid treatment, PO antihistamines, and pulse PO steroids, if necessary.
   3. ≥Grade 3 nausea and/or vomiting and ≥ Grade 3 diarrhea that has resolved to < Grade 3 within 72 hours of optimal antiemetic and/ or antidiarrheal treatment. (All patients should receive optimal antiemetic and/ or antidiarrheal treatment according to standard of care. An optimal antiemetic regimen is defined as one that employs a 5-hydroxytyptamine 3 serotonin receptor (5-HT3) antagonist and a corticosteroid given in standard doses and according to standard schedules).
   4. Transient Grade 3 fatigue (≤ 72 hours)
   5. Asymptomatic lipase elevation (< Grade 4) in the absence of significant amylase elevation (< Grade 3) considered not dose limiting by the treating physician.
   6. Asymptomatic amylase elevation (< Grade 4) in the absence of significant lipase elevation (< Grade 3) considered not dose limiting by the treating physician.
   7. Asymptomatic Grade 3 elevation of a single liver enzyme (AST or ALT) in the absence of significant bilirubin elevation (< Grade 3) considered not dose limiting by the treating physician.
7. Inability to administer at least 75% of planned doses of study drug within cycle 1 of TAK-659 (Cycle 2 of the study) due to treatment-related toxicity.
8. Other TAK-659-related non-hematologic toxicities Grade 2 or greater that, in the opinion of the investigator, require discontinuation of therapy with TAK-659.

Supplemental 4: Phase I Escalation Scheme and Determination of MTD

Patients will be enrolled to dose level 1 initially (TAK-659 60 mg). Dose escalation will proceed up to 80mg for dose level 2, and 100 mg for level 3. The table below summarizes the dose levels. A dose level -1 is included in the event that level 1 is determined to be too toxic.


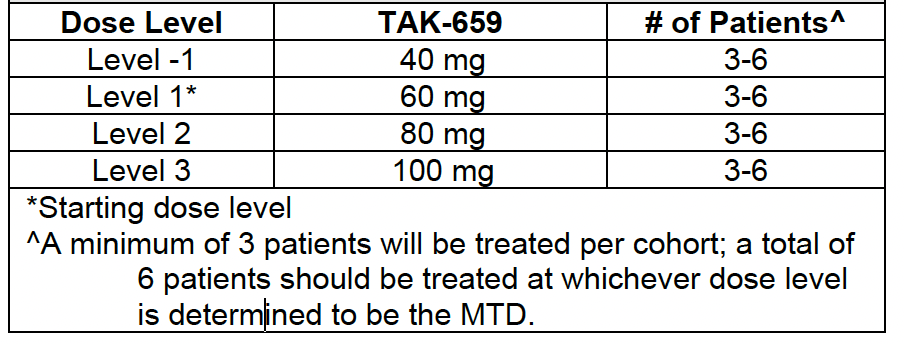


A standard “3+3” dose escalation design will be utilized. Initially, 3 patients will be enrolled at the starting dose (level 1), after which enrollment will be temporarily suspended until all 3 patients complete the DLT evaluation period (defined as cycle 2 of the study (the first 21-day cycle of TAK-659)). Once all 3 patients complete the DLT period and toxicity data has been submitted, the Data and Safety Monitoring Committee (DSMC) will review the data and confirm the presence or absence of any DLTs. The following rules will be used at each dose level to determine whether or not to proceed to the next dose level:

- If 0 of 3 patients at a given dose level experience a DLT (defined below), then escalation will proceed to the next dose level.
- If 2 or 3 of 3 patients at a given dose level experience a DLT, then one of the following must occur:
  - If this happens at level 1, de-escalation to level -1 will occur.
  - If this happens at level 2 or beyond, the previous level will be declared the maximum tolerated dose (MTD).
  - If this happens at level -1, the study will be closed to further accrual and the regimen of TAK-659 + R-CHOP will be considered too toxic at any dose.
- If 1 of 3 patients at a given dose level experiences a DLT, then an additional 3 slots will be added (for a total of 6 patients at that level):
  - If 1 of 6 total experiences a DLT, then escalation will proceed to the next level.
  - If ≥ 2 of 6 total experience a DLT, the previous level will be declared the MTD.

NOTE: Whichever dose level is declared the MTD must have 6 total patients treated at that level. For example, if 3 patients are treated at level 2 and 0 patients experience DLT, escalation would then proceed to level 3. However, if ≥ 2 patients at level 3 experience DLT, enrollment to level 2 would need to be re-opened to enroll an additional 3 patients at that level (with 0 or 1 DLT observed in 6 total patients) in order to declare level 2 the MTD.
